# Physician-patient communication about overactive bladder: Results of an observational sociolinguistic study

**DOI:** 10.1371/journal.pone.0186122

**Published:** 2017-11-15

**Authors:** Steven R. Hahn, Pamela Bradt, Kathleen A. Hewett, Daniel B. Ng

**Affiliations:** 1 Albert Einstein College of Medicine, Bronx, New York, United States of America; 2 Jacobi Medical Center, Bronx, New York, United States of America; 3 Medical Affairs, Americas Astellas Pharma Global Development, Inc., Northbrook, Illinois; 4 Ogilvy CommonHealth Behavioral Insights, Parsippany, New Jersey, United States of America; Carolina Urologic Research Center, UNITED STATES

## Abstract

**Introduction:**

Overactive bladder (OAB) and urinary incontinence are common problems that have significant impact on quality of life (QOL). Less than half of sufferers seek help from their physicians; many who do are dissatisfied with treatment and their physicians’ understanding of their problems. Little is known about the sociolinguistic characteristics of physician-patient communication about OAB in community practice.

**Methods:**

An IRB-approved observational sociolinguistic study of dialogues between patients with OAB and treating physicians was conducted. Study design included semi-structured post-visit interviews, post-visit questionnaires, and follow-up phone calls. Conversations were analyzed using techniques from interactional sociolinguistics.

**Results:**

Communication was physician- rather than patient-centered. Physicians spoke the majority of words and 83% of questions were closed-ended. The impact of OAB on QOL and concerns about and adherence to treatment were infrequently addressed by physicians, who were poorly aligned with patients in their understanding. These topics were addressed more frequently when open-ended questions successfully eliciting elaborated responses were used in ask-tell-ask or ask-tell sequences.

**Discussion:**

Clinical dialogue around OAB is physician-centered; topics critical to managing OAB are infrequently and inadequately addressed. The use of patient-centered communication is correlated with more discussion of critical topics, and thus, more effective management of OAB.

## Introduction

Overactive bladder (OAB) and urinary incontinence are common problems that affect quality of life (QOL). Urinary incontinence affects 45% of women between the ages of 30 and 90, ranging from 28% of women in their thirties to 55% of women aged 80 to 90 years.[1] The prevalence of severe incontinence ranges from 8% to 33% across this age span.[1] Incontinence has a significant impact on QOL[2] contributing to depression,[3, 4] social isolation,[5] falls,[6] sexual dysfunction and self-esteem,[7] hospitalizations and nursing home admissions.[8] Despite these consequences, less than half of patients seek treatment for their lower urinary tract problems,[9, 10] and those that do talk to their physicians delay for up to a year from symptom onset.[7] Reasons cited for not seeking help include the belief that lower urinary tract symptoms and incontinence are natural consequences of aging, untreatable, and hygiene problems rather than medical problems.[11] Most patients value communication with their physicians and hope the physicians will initiate the conversation, but they rarely do,[7, 12] and most patients remain unsatisfied with the treatment offered and the self-management strategies they resort to.[7] Patients are also dissatisfied with their communication with physicians about OAB and incontinence and many feel that their healthcare providers do not understand their experience or take it seriously.[12] This description of clinical communication in OAB and incontinence is consistent with observations of physician-patient communication in community practice in several different therapeutic areas including migraine,[13, 14] glaucoma,[15, 16] and female sexual dysfunction[17] in which sociolinguistic analysis of video-recorded clinical encounters revealed homogeneously physician-centered communication. In these studies, physicians failed to elicit a narrative of the patient’s experience with their symptoms, functional impairment, and treatment and consequently failed to recognize the need for preventive medication,[13, 14] adherence to treatment, [15, 16] and presence of disorder,[17] respectively. Targeted educational interventions designed to enhance patient-centered communication were effective in changing physicians’ communication strategies and improving the detection of the need for preventive medication in migraine[13] and detection and discussion about nonadherence in glaucoma management.[16] The current study reports findings of a sociolinguistic analysis of video-recorded encounters between community physicians and patients with OAB.

## Materials and methods

### Study design

This was an observational study of naturally-occurring dialogues between patients with OAB and their physicians. It was conducted in April-May 2012, with the oversight of the Independent Investigational Review Board, Inc., and designed in compliance with the Health Insurance Portability and Accountability Act of 1996 (HIPAA). All recorded participants provided informed written consent.

### Physician and patient selection

Potential physicians (n = 1000) in community-based practices with a substantial population of OAB patients (as determined by prescribing decile on a sponsor-provided list) were invited to participate in a physician-patient communication study of patients diagnosed with OAB. The sponsor was not identified to participants and participant identities were not revealed to the sponsor.

Physicians were required to be board-certified, in practice 2–30 years, full-time, in an office-based practice, spend at least 80% of time in direct care, conduct at least 75% of patient discussions in English, not have participated in market research regarding management of OAB in the past three months, and not be employed or have an immediate family member employed by a pharmaceutical company, government regulatory agency, or as a consultant to any pharmaceutical or market research agency. During an average month, primary care physicians (PCPs) were required to see at least 400 patients, and urologists, gynecologists, and urogynecologists were required to see at least 300 patients. Additionally, during an average month, PCPs, gynecologists, and urogynecologists were required to see at least five patients to discuss OAB and write at least three OAB prescriptions, and urologists were required to see at least eight patients and write at least six prescriptions. Of the last 30 OAB prescriptions they wrote, at least 80% were required to be new or refills of prescriptions that they had initiated, rather than refills of prescriptions that had been initiated by others. Of the last 30 patients who presented with OAB symptoms that they treated with prescription medication, at least 5% were required to be treated with solifenacin succinate. A total of 21 (0.021%) physicians responded, of these 17 (0.017%) met the criteria and agreed to participate, which was a typical response rate for this methodology and fell within the goal range.[13–19] PCPs (n = 7), urologists (n = 5), gynecologists (n = 3), and urogynecologists (n = 2) were enrolled.

Patients were recruited on the day of fieldwork. One member of a team of ethnographic researchers conducted a day of research at each practice. Physicians and any other recorded participants provided informed written consent. A staff member at each office invited patients with a regularly scheduled appointment that day and met the criteria, to participate in a study regarding physician-patient communication. Patients were required to have a physician-identified pre-existing diagnosis of OAB, with a scheduled visit to discuss OAB treatment, be fluent in English, not be cognitively impaired as determined by their physician, and be either a newly diagnosed (within the last six months) female patient, or an established (six months or more) male or female patient. Patients who expressed interest met with the researcher to receive study information and sign consent.

### Data collection

One visit per patient was video- and audio-recorded without the researcher present. Following the visit, patients participated in 20-minute semi-structured interviews and completed questionnaires. Physicians participated in interviews at the end of the day, in which they answered questions about their beliefs and practices (10–15 minutes) and about each participating patient (20 minutes each). Physicians were able to use medical records to aid recall, but these were not shared with researchers. Physicians also completed written questionnaires about each patient. Physicians and patients were asked similar questions in the interviews and questionnaires to assess alignment. Approximately one month after the visit, patients were interviewed again by telephone. All participants received a small compensation for participation in the research.

Overall, 56 patients were approached, all were consented, and all had their visits recorded, but some of these visits did not meet the eligibility requirements resulting in 14 patients being excluded. Of these, three did not have a diagnosis of OAB, two had limited discussion of OAB treatment, and nine established patients were excluded to achieve an approximately even distribution of newly diagnosed and established patients. The remaining 42 visits contributed to the findings, which is a typical sample size for discourse analysis.[13–19] Each of the physicians interacted with at least one patient for the final sample; none contributed more than four, and the average was two to three patients per physician.

### Sociolinguistic analysis

All visits and interviews were transcribed using audio recordings. Video recordings allowed for transcript quality control and assessment of non-verbal cues (e.g. nodding). Interactional sociolinguistic methods were used. These include categorizing discourse at the word and topic level, flow of information, and the roles of those speaking.[20, 21] An analytic team of social scientists and clinicians reviewed a sample of seven transcripts and identified trends. With these hypotheses, analyses were developed and conducted on the full dataset, including the sample, by the social scientists, to assess the validity and pervasiveness of these trends. The content of physician-patient discussion was compared to the corresponding post-visit interviews to identify gaps in communication.

Analyses followed and built upon previously established sociolinguistic techniques.[13–21] These included, but were not limited to, quantifying the percentage of words spoken by physicians and dividing dialogue into unique physician “asks” (i.e. questions) and “tells” (i.e. statements). “Small talk” and instructions during physical exams were excluded from the analysis. Listener responses (i.e. backchanneling), such as “uh-huh” and requests for clarification, were not considered unique and were treated as part of the corresponding ask or tell. Analyses also included categorizing the type (e.g. open-ended, short-answer, closed-ended) and “success” (open-ended question that elicits an elaborated response from the patient) of each ask sequence. The “success” of ask sequences is a newly defined measure. Recognizing that patients may have to be asked more than once to elicit an elaborated rather than short answer, success in eliciting an elaborated answer was tracked over two related questions in a sequence. When ask sequences are followed by a related tell, it forms an ask-tell sequence, and when an ask-tell sequence is followed by a second ask to assess the impact of the tell, it forms an ask-tell-ask sequence. We enhanced previously used strategies for identifying ask-tell-ask sequences which depended on the elements occurring in temporal proximity to account for dialogue where the tell and second ask may occur remotely from the initial ask.[13–17] We therefore tracked asks and tells by content theme across the entire encounter and identified ask-tell-ask, ask-tell, and tell ask-sequences across the duration of the encounter.

Presence, robustness, and linguistic characteristics of discussions about QOL, treatment concerns, and adherence were also assessed. Treatment changes were captured, by evaluation of visit dialogue and physician post-visit interview. “Alignment” between physicians and patients about the impact of OAB symptoms on QOL was evaluated by comparing responses to similar questions in post-visit assessments. To insure consistency in scoring, all of the ask-tell-ask sequence analysis was conducted by one of the authors (KAH) and any instances of uncertainty regarding characterization of sequences was resolved by consensus with a second author (SRH). Descriptive statistics were applied to components of interest.

## Results

### Physician and patient characteristics

A total of 17 physicians and 42 patients were enrolled in the study Tables 1 and 2. Of the patients, 14 attended visits with their PCP, 11 with their urologist, 10 with their gynecologist, and seven with their urogynecologist.

**Table 1:** Patient demographic information (n = 42).

Characteristic	Value
**Age (y)**	
Mean ± SD (standard deviation)	58 ± 17.88
Range	23–85
**Female gender, n (%)**	39 (93)
**Race/ethnicity, n (%)**	
Caucasian	31 (73)
African-American	7 (17)
Asian (including Indian subcontinent)	2 (5)
Hispanic	2 (5)
**Insurance coverage, n (%)**	41 (98)
**Prescription coverage, n (%)**	41 (98)
**Length of physician-patient relationship, n (%)**	
First visit	1 (3)
<1 year	14 (33)
1–3 years	8 (19)
3–5 years	3 (7)
>5 years	16 (38)
**Visit frequency, n (%)**	
First visit	1 (3)
More than once per month	7 (17)
Once per month	6 (14)
Every 2–3 months	14 (33)
Every 4 months	2 (5)
Every 6 months	5 (12)
Other	7 (16)
**Established vs. newly diagnosed, n (%)**	
Established (diagnosed 6 months ago or longer)	25 (60)
Newly diagnosed (diagnosed within the past 6 months)	17 (40)
**Urgency vs. both urgency and stress incontinence, n (%)**	
Urgency only	21 (50)
Both urgency and stress incontinence	21 (50)

**Table 2:** Physician demographic information (n = 17).

Characteristic	Value
**Specialty, n (%)**	
Primary care	7 (41)
Urology	5 (29)
Gynecology	3 (18)
Urogynecology	2 (12)
**Years in practice (y)**	
Mean ± SD (standard deviation)	19 ± 8.76
Range	4–30
**Male gender, n (%)**	14 (82)
**Location, n (%)**	
California	3 (17)
Florida	2 (12)
Illinois	2 (12)
Michigan	1 (6)
New Jersey	4 (23)
New York	1 (6)
Ohio	2 (12)
South Carolina	1 (6)
Texas	1 (6)

Physicians had an average of 19 years in practice and were predominantly male (14/17, 82%), Table 2. Patients were 93% female, with an average age of 58 years; all but one patient had seen the physician previously, Table 1. The average visit lasted 9.87 minutes (standard deviation [SD], 6.05; median, 7.75 minutes).

### Patient-centered communication techniques

Physician-patient dialogue was predominantly physician-centered. Physicians spoke 62% of the words and 83% of physician’s questions were closed-ended. Physicians (82%, n = 14) asked at least one open-ended question in 67% (n = 28) of visits. Physicians (76%, n = 13) used successful ask sequences eliciting more than a yes/no or short response in 52% (n = 22) of visits, and these constituted an average of 16% of all ask sequences, Table 3. Although physicians (100%, n = 17) used at least one ask-tell sequence in 93% (n = 39) of visits, physicians (59%, n = 10) used at least one ask-tell-ask sequence in 33% (n = 14) of visits and they (12%, n = 2) used more than one ask-tell-ask sequence in 7% (n = 3) of visits. Physicians (18%, n = 3) used ask-tell-ask sequences that included an open-ended question in 7% (n = 3) of visits and they (12%, n = 2) used ask-tell-ask sequences that included a successful ask sequence in 5% (n = 2) of visits, Table 3.

**Table 3:** Example of ask-tell-ask sequence including successful ask sequence.

Component	Dialogue
**First Ask** (also a successful ask sequence i.e. open-ended question with elaborated patient response)	**PHYSICIAN**: Where are you currently in kind of your treatment and how are you feeling? **PATIENT**: I’m starting to see some improvement. **PHYSICIAN**: Okay. **PATIENT**: Umm, I don’t get up at night. **PHYSICIAN**: That’s good. **PATIENT**: That’s good. I don’t have to get up at night anymore. And when I feel I have to go to the bathroom, if I go, I make it there. **PHYSICIAN**: That’s a plus. **PATIENT**: That’s a plus. And, umm, just like coughing or sneezing, I have problems.
**Tell**	**PHYSICIAN**: Okay. That’s a kind of a different issue, but a lot of times, umm, the medications and the stimulator will make that better along with some other muscle exercises. **PATIENT**: Yeah. **PHYSICIAN**: You remember the old Kegel— **PATIENT**: Kegel. **PHYSICIAN**: Exercises from long ago. There are higher—there’s sort of a high tech version of that known as, PMR or Pelvic Muscle Re-education. Umm, and that can be done very simply in the office. Again, a k—Medicare-covered type of benefit. And where we sort of help you with helping your muscles—making sure that you’re tightening all the right muscles. And when you’re good at tightening those muscles, of course, you can do them anywhere you want. You can do them while you’re watching television or you’re sitting in a traffic light or doing whatever. So, that may be something that at some point in time we add that back to your program to make you even better than you are now…
**Second Ask**	**PHYSICIAN**: So does that sound like something you might want to pursue at some point in time— **PATIENT**: Yes. Yes.

Despite conversations that comprised predominantly closed-ended questions, patients often gave elaborated responses, i.e. they went beyond the asked for “yes” or “no” and responded as though they had been asked an open-ended question. On average, there were 5.5 patient-elaborated responses per encounter and at least one patient-elaborated response in 95% of encounters.

### Topics discussed and visit outcomes

Several key topics were rarely discussed during visits. Physicians (71%, n = 12) discussed the impact of OAB on QOL in 40% (n = 17) of visits and physicians (29%, n = 5) brought it up in 19% (n = 8) of visits. However, in response to open-ended and structured questions during post-visit interviews, most patients (93%, n = 39) said that their urinary problems had a significant impact on QOL, and most physician-patient pairs were not aligned on the impact their symptoms had on QOL (82%, 33 of 40). Physicians (47%, n = 8) changed OAB treatment in 33% (n = 14) of visits.

Physicians (41%, n = 7) attempted to allay patients’ concerns about OAB treatments in 24% (n = 10) of visits. Physicians (41%, n = 7) discussed adherence to OAB treatment in 17% (n = 7) of visits. However, during post-visit interviews, one-third of patients prescribed medication admitted to nonadherence (37%, 14 of 38). Physicians believed that 63% (24 of 38) of their patients on medications always followed recommendations and that 37% (14 of 38) sometimes did not. An equal proportion 63% (24 of 38) of patients described themselves as never skipping medications and 37% (14 of 38) acknowledged that sometimes they skip or forget. However, there was no association between the patients’ self-report and physicians’ perceptions of patients’ adherence behavior. Just over half (53% n = 19) of physician-patient pairs were aligned on skipping or forgetting medication (N = 38, Kappa = -0.018, *P* = 0.912). Physicians more often identified when patients stopped medication on their own; they classified 85% of the 35 patients who admitted to stopping as sometimes nonadherent but this association was not statistically significant (N = 35, Kappa = .231, *P* = .139).

### Association between communication characteristics and patient-reported outcomes

**Fig 1:**
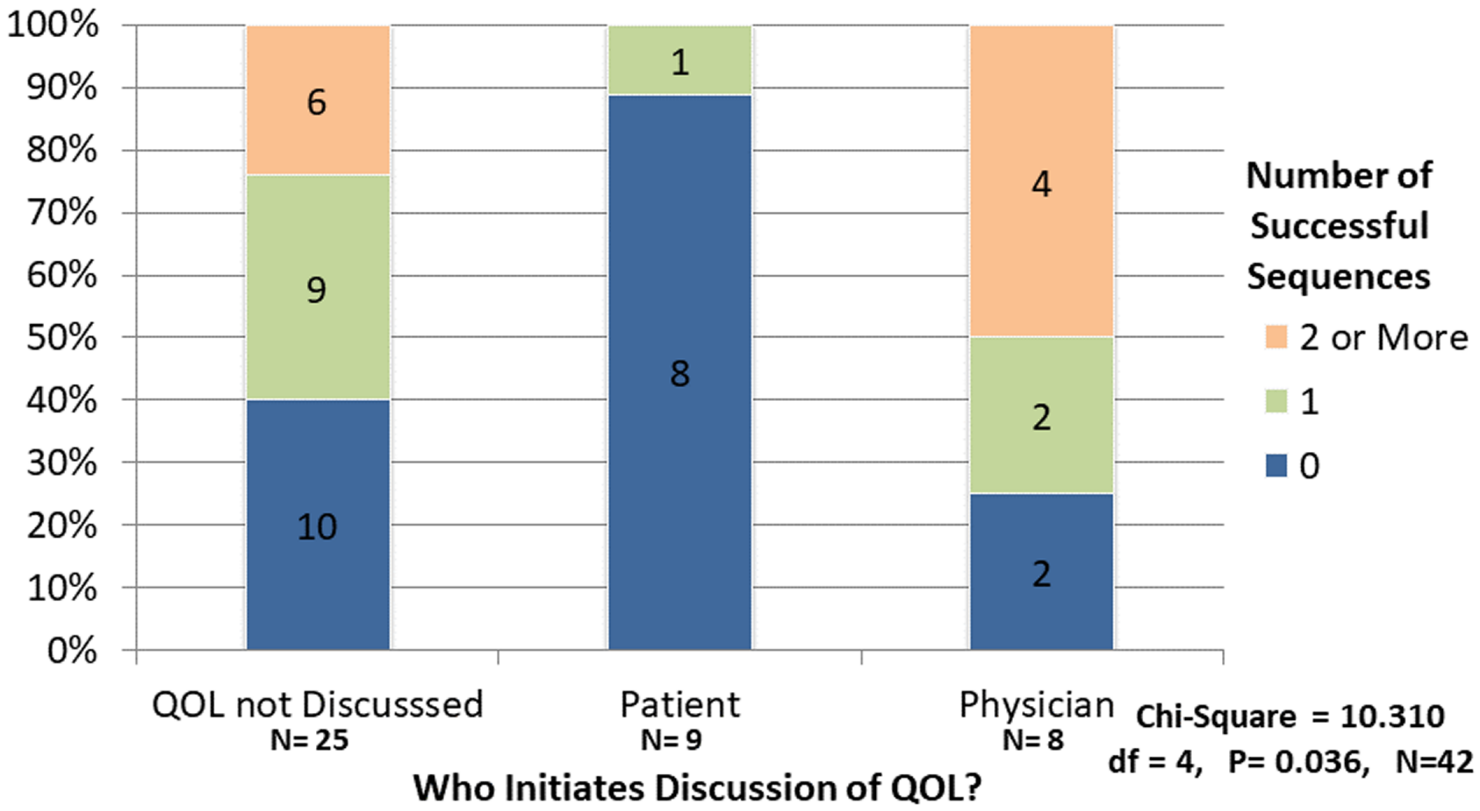
Who initiates discussion of QOL and number of successful ask sequences.

QOL discussions were more likely to be initiated by the physician and never had to be initiated by the patient when the physician used two or more successful ask sequences, Fig 1. Most patient-initiated discussion of QOL occurred when physicians did not use any successful ask sequences. Fewer encounters with 2 two or more successful ask sequences left QOL unaddressed compared to those with one or no successful ask sequences.

**Fig 2:**
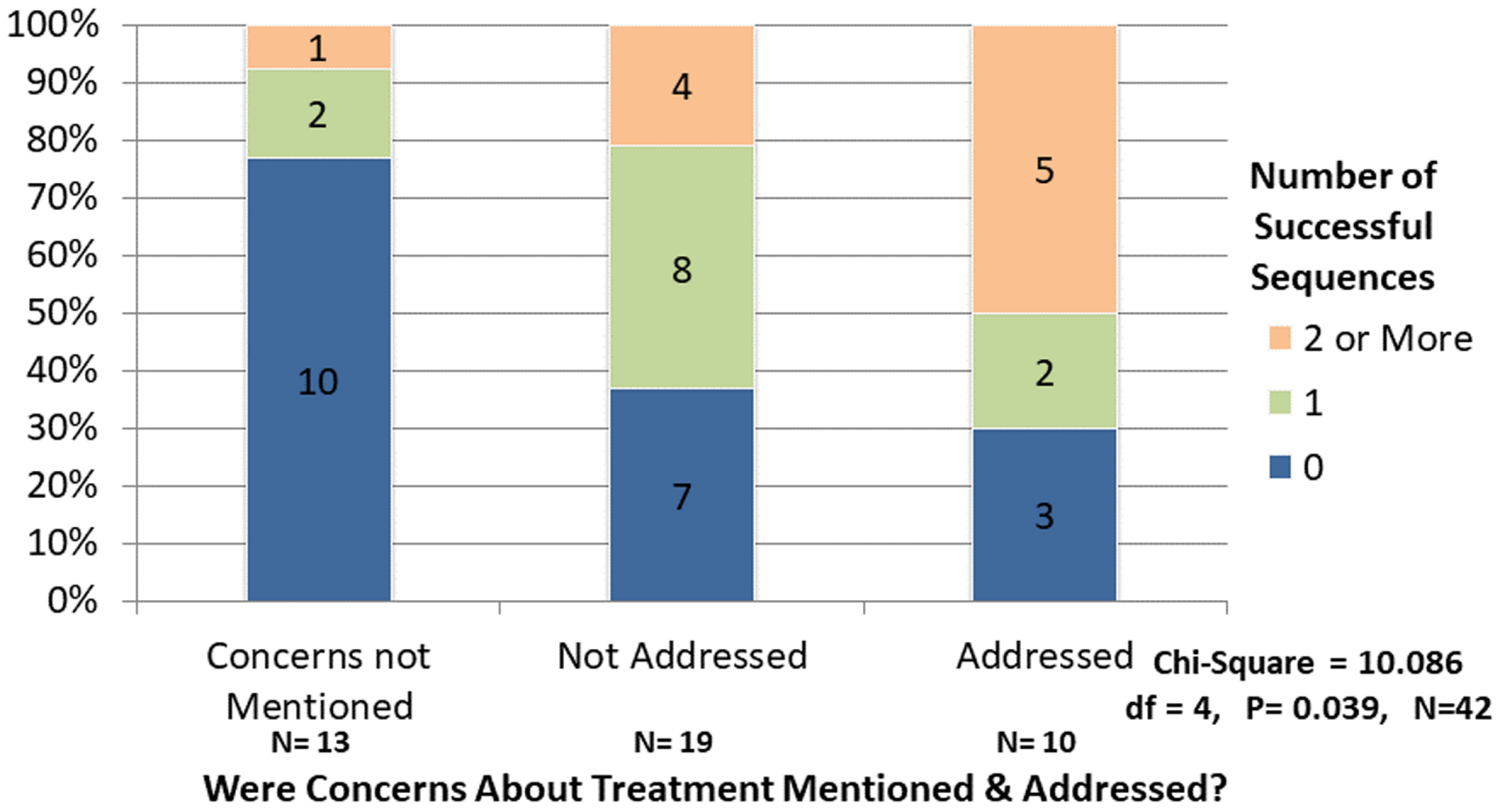
Discussion of concerns about treatment and number of successful ask sequences.

Physicians addressed patient concerns about treatment in 50% of visits with two or more successful ask sequences compared to only 20% in those with none and 30% of those with one sequence, Fig 2. Concerns about treatment went unmentioned in the majority (77%) of encounters with no successful sequences and were more frequently mentioned, but left unaddressed, in encounters when less than two successful ask sequences were employed.

In visits where physicians changed treatments, physicians used more ask-tell-ask sequences (0.64 vs. 0.32, *P* = 0.165) and more ask-tell sequences (2.93 vs. 2.29, *P* = 0.236) and patients had more patient elaborated responses (7.50 vs. 4.54, *P* = 0.062). Treatments were changed in three of three of the encounters that included two or three ask-tell-ask sequences compared to 18% (2/11) of those with only one and 32% (9/28) of those with no ask-tell-ask sequences (*P* = 0.028) Physicians were more likely to change treatment for patients who were dissatisfied with their treatment (86%, 6 of 7) compared to those who were satisfied (22%, 4 of 18) or extremely satisfied (0%, 0 of 6; *P* = 0.002). Medications were changed in 60% (6 of 10) of visits where the patient expressed their dissatisfaction during the visit compared to 25% (8 of 32) of visits where dissatisfaction was not expressed (*P* = 0.040)

## Discussion

Our observations demonstrate that clinical dialogue between patients with OAB and their physicians is physician-centered. Physicians spoke most of the words and asked closed-ended questions that do not invite the patient to tell the story of their illness or its treatment. In a condition such as OAB, where impaired QOL is the most significant consequence, QOL was only discussed in 40% of encounters and the conversation was only initiated by physicians in one out of five encounters. However, post-visit, 93% of the patients said that OAB had a significant impact on their lives. The lack of discussion around QOL was not because physicians were already familiar with the impact of OAB; in fact, they were less likely to be in alignment with their established patients than they were with newly diagnosed patients. Similar deficits were observed in physicians’ attention to treatment adherence. About a third of patients admitted to nonadherence, but adherence was only addressed in 17% of encounters and physicians had no better than chance probability of identifying patients who had skipped or stopped medication. One area in which physicians were attentive to their patients’ experience was their response to dissatisfaction with treatment. Physicians were more likely to change medications when patients were dissatisfied with their treatment.

The patterns of communication observed in this study are consistent with previous in-office dialogue studies in migraine[13, 14], glaucoma[15, 16] and female sexual dysfunction [17] demonstrating physician-centered rather than patient-centered communication. In all of these studies, which employed the same sociolinguistic analyses, physicians asked between 0.9 and 1.1 questions per minute, 91% to 94% were closed-ended and fewer than 10% of encounters addressed functional impairment, even for conditions as dramatically impairing as migraine headache. As a consequence physicians failed, respectively, to detect the need for or prescribe migraine preventive medication[13, 14], detect nonadherence to glaucoma medication and patients’ suboptimal perceived need for treatment[15, 16] and to explore beyond establishing the presence of a problem in women with sexual dysfunction[17].

In this study, we employed several new sociolinguistic analytic strategies for the first time. In previous studies we enumerated the frequency and topic of open vs. closed-ended questions and the use of ask-tell-ask, ask-tell and tell-ask sequences when the elements were temporally contiguous [16]. In this study our goal was to identify ask-tell-ask, ask-tell and tell-ask sequences even when the elements might be dispersed throughout the encounter and we achieved this goal by tracking asks and tells by content theme across the entire encounter. We also wanted to classify the “success” of physicians’ asks, defined as using an open-ended question and receiving an elaborated rather than yes/no or short answer. Because patients often need to be asked open-ended questions twice before they actually respond with an elaborated answer, we determined whether an ask was successful over two sequential questions containing an open-ended question. Recognizing that patients may give an elaborated answer to a physician’s yes/no question, we classified these sequences as “patient elaborated responses.”

The frequency of patient-centered communication in the observed dialogue was very low and therefore the associations observed between patient-centered communication and visit-related outcomes were modest. A number of theoretically important patient-centered communication skills were essentially absent in this study including dialogue about the patient’s beliefs in the importance of prescribed treatments and use of a normalizing and shared decision-making strategy for detecting nonadherence. However, the newly defined measure of “successful ask sequences” was associated with more effective communication about the important domains of QOL and concerns about treatment. The low frequency of patient-centered communication skills documented in this study can serve as a baseline for future efforts to enhance communication and study the effect on visit outcomes.

An obvious limitation of this study is the small sample size, a consequence of the cost of real-world video recording and labor intensive sociolinguistic analysis. However, our sample of 42 encounters is within the standard and accepted range for studies of this kind.[13–19] The relative homogeneity of observed behavior and descriptive power of the classification allows for reliable characterization of the most important characteristics of the observed behavior. Nevertheless, the study does not have the power to confidently assess the significance of many associations between communication, patient and physician characteristics, and outcomes that are undoubtedly important. It is also not possible to assess the range of physician performance or the contribution of physician characteristics when each physician contributed only a few patients. Differences between PCPs and specialists were investigated, but none proved significant.

## Conclusion

Structured sociolinguistic analysis of clinical dialogue between established community physicians and their patients with OAB is characterized by physician-centered rather than patient-centered communication. Patients and physicians are poorly aligned in their understanding of the impact that OAB has on QOL, the extent of nonadherence to treatment, and the need to address concerns about treatment. Communication around OAB is similar in this respect to clinical communication studied with similar methods in other therapeutic areas such as migraine, glaucoma, and sexual dysfunction. The associations that were observed between the frequency of successful ask sequences and important domains including QOL and dialogue around concerns about treatment suggest that this new metric may be a helpful addition to methods of assessing the effectiveness of clinical communication. This study should be helpful as a benchmark in future efforts to improve communication and care in patients with OAB.

## Supporting information

S1 Text Patient follow-up call discussion guide. (DOCX)

S2 Text Post-visit patient discussion guide. (DOCX)

S3 Text Post-visit patient questionnaire. (DOCX)

S4 Text Post-visit physician discussion guide. (DOCX)

S5 Text Post-visit physician patient-specific questionnaire. (DOCX)

S1 Data Physician and patient sociolinguistic study data. (XLSX)

## References

[1] Melville JL, Katon W, Delaney K, Newton K. Urinary incontinence in US women: a population-based study. *Arch Intern Med* 2005 3 14;165(5):537–42. doi: 10.1001/archinte.165.5.537 15767530 10.1001/archinte.165.5.537

[2] Huang AJ, Brown JS, Kanaya AM, Creasman JM, Ragins AI, Van Den Eeden SK et al Quality-of-life impact and treatment of urinary incontinence in ethnically diverse older women. *Arch Intern Med* 2006 10 9;166(18):2000–6. doi: 10.1001/archinte.166.18.2000 17030834 10.1001/archinte.166.18.2000

[3] Dugan E, Cohen SJ, Bland DR, Preisser JS, Davis CC, Suggs PK et al The association of depressive symptoms and urinary incontinence among older adults. *J Am Geriatr Soc* 2000 4;48(4):413–6. 10798468 10.1111/j.1532-5415.2000.tb04699.x

[4] Avery JC, Stocks NP, Duggan P, Braunack-Mayer AJ, Taylor AW, Goldney RD et al Identifying the quality of life effects of urinary incontinence with depression in an Australian population. *BMC Urol* 2013;13:11 doi: 10.1186/1471-2490-13-11 23413970 10.1186/1471-2490-13-11 PMC3585815

[5] Yip SO, Dick MA, McPencow AM, Martin DK, Ciarleglio MM, Erekson EA. The association between urinary and fecal incontinence and social isolation in older women. *Am J Obstet Gynecol* 2013 2;208(2):146–7. doi: 10.1016/j.ajog.2012.11.010 23159696 10.1016/j.ajog.2012.11.010 PMC3715999

[6] Brown JS, Vittinghoff E, Wyman JF, Stone KL, Nevitt MC, Ensrud KE et al Urinary incontinence: does it increase risk for falls and fractures? Study of Osteoporotic Fractures Research Group. *J Am Geriatr Soc* 2000 7;48(7):721–5. 10894308 10.1111/j.1532-5415.2000.tb04744.x

[7] Dmochowski RR, Newman DK. Impact of overactive bladder on women in the United States: results of a national survey. *Curr Med Res Opin* 2007 1;23(1):65–76. doi: 10.1185/030079907X159533 17257467 10.1185/030079907X159533

[8] Thom DH, Haan MN, Van Den Eeden SK. Medically recognized urinary incontinence and risks of hospitalization, nursing home admission and mortality. *Age Ageing* 1997 9;26(5):367–74. 9351481 10.1093/ageing/26.5.367

[9] Huang AJ, Thom DH, Kanaya AM, Wassel-Fyr CL, Van Den Eeden SK, Ragins AI et al Urinary incontinence and pelvic floor dysfunction in Asian-American women. *Am J Obstet Gynecol* 2006 11;195(5):1331–7. doi: 10.1016/j.ajog.2006.03.052 16643821 10.1016/j.ajog.2006.03.052 PMC1630451

[10] Benner JS, Becker R, Fanning K, Jumadilova Z, Bavendam T, Brubaker L. Bother related to bladder control and health care seeking behavior in adults in the United States. *J Urol* 2009 6;181(6):2591–8. doi: 10.1016/j.juro.2009.02.018 19375096 10.1016/j.juro.2009.02.018

[11] Newman DK. Talking to patients about bladder control problems. *Nurse Pract* 2009 12;34(12):33–45. doi: 10.1097/01.NPR.0000365126.88904.67 19952586 10.1097/01.NPR.0000365126.88904.67

[12] Filipetto FA, Fulda KG, Holthusen AE, McKeithen TM, McFadden P. The patient perspective on overactive bladder: a mixed-methods needs assessment. *BMC Fam Pract* 2014;15:96 doi: 10.1186/1471-2296-15-96 24885491 10.1186/1471-2296-15-96 PMC4030445

[13] Hahn SR, Lipton RB, Sheftell FD, Cady RK, Eagan CA, Simons SE et al Healthcare provider-patient communication and migraine assessment: results of the American Migraine Communication Study, phase II. *Curr Med Res Opin* 2008 6;24(6):1711–8. doi: 10.1185/03007990802122388 18471346 10.1185/03007990802122388

[14] Lipton RB, Hahn SR, Cady RK, Brandes JL, Simons SE, Bain PA et al In office discussion of migraine: Results from the American Migraine Communication Study. *JGIM* 2008;23(8):1145–51. doi: 10.1007/s11606-008-0591-3 18459012 10.1007/s11606-008-0591-3 PMC2517978

[15] Friedman DS, Hahn SR, Quigley HA, Kotak S, Kim E, Onofrey M et al Doctor-patient communication in glaucoma care: analysis of videotaped encounters in community-based office practice. *Ophthalmology* 2009 12;116(12):2277–85. doi: 10.1016/j.ophtha.2009.04.052 19744715 10.1016/j.ophtha.2009.04.052

[16] Hahn SR, Friedman DS, Quigley HA, Kotak S, Kim E, Onofrey M et al Effect of patient-centered communication training on discussion and detection of nonadherence in glaucoma. *Ophthalmology* 2010 7;117(7):1339–47. doi: 10.1016/j.ophtha.2009.11.026 20207417 10.1016/j.ophtha.2009.11.026

[17] Hahn SR, Parish SJ, Onofrey M, Wysoki S, Kingsberg S, Perelman MA. Doctor-patient communication about the impact of distressing decreased sexual desire on women's quality of life. 2010.

[18] Davidson B, Blum D, Cella D, Hamilton H, Nail L, Waltzman R. Communicating about chemotherapy-induced anemia. *J Support Oncol* 2007 1;5(1):36–40, 46. 17265785

[19] Davidson B, Vogel V, Wickerham L. Oncologist-patient discussion of adjuvant hormonal therapy in breast cancer: results of a linguistic study focusing on adherence and persistence to therapy. *J Support Oncol* 2007 3;5(3):139–43. 17410813

[20] Gumperz JJ. On interactional sociolinguistic method In: Sarangi S, Roberts C, editors. *Talk*, *work and institutional order*. *Discourse in medical*, *mediation and management setting*.Berlin: Mouton de Gruyter; 1999 p. 453–71.

[21] Hamilton HE. Symptoms and signs in particular: the influence of the medical concern on the shape of physician-patient talk. *Commun Med* 2004;1(1):59–70. doi: 10.1515/come.2004.006 16808689 10.1515/come.2004.006

