# Supplementary material for: Physician-patient communication about overactive bladder: Results of an observational sociolinguistic study
Source: PLoS One. 2017 Nov 15;12(11):e0186122. doi: 10.1371/journal.pone.0186122 (PMC5687746; doi:10.1371/journal.pone.0186122)
Supplement: S1 Text — (DOCX) [file pone.0186122.s001.docx]

**Ogilvy CommonHealth Insights & Analytics**

**OAB Patient Follow-up Call Discussion Guide**

*Thank you for taking the time to speak with us on the phone today. This call is in relation to the communications study you participated in approximately a month ago to discuss “bladder issues” issues with your doctor. We are following up to ask you some questions relating to that visit and your health in general. All of your answers are private, and will not be shared with your doctor.*

1. Just for our records, please tell me your name, age, and the doctor you visited with on the day we recorded you.

Name: __________________________

Age: __________________________

Physician: __________________________

1. During that visit, what do you remember discussing about your bladder issues? *(Keep this general – as a starter to refresh their memory about the visit and moving forward, use the term they use for their condition.)* ________________________________________________________________________________________________________________________________________________________________________________________________________________________
2. Was there anything you wanted to discuss during that visit, but did not?

⁪ Yes ⁪ No

- 1. *If yes, ask:* What, if anything, have you done to answer your questions since the visit? *(Probe for* c*alling the office to ask, researching online, asking a friend, etc.)*

________________________________________________________________________________________________________________________________________________________________________________________________________________________

1. Thinking back to the visit where we recorded you, what thoughts, concerns, or emotions stand out for you during your discussion with the doctor?
2. Were these concerns/emotions addressed by the doctor?

________________________________________________________________________________________________________________________________________________________________________________________________________________________

b. How do you feel about these concerns/emotions now, a few weeks later?

________________________________________________________________________________________________________________________________________________________________________________________________________________________

1. During the visit where we recorded you, did your doctor write a prescription for a medication or provide samples for your [use patient term]?

⁪ Yes ⁪ No Specify: [Samples] v. [Rx]

1. *If yes*, have you filled that medication prescription and/or begun taking those samples? Please explain.

________________________________________________________________________________________________________________________________________________________________________________________________________________________

________________________________________________________________________________________________________________________________________________

1. Have there been any changes in your [use patient term] symptoms since the visit where we recorded you?

⁪ Yes ⁪ No

- 1. *If yes*, have they been improving or worsening?
     1. *If symptoms are improving, ask:*  How satisfied are you with how well the medication has been working?
     2. *If symptoms are worsening, ask:* Have you told your doctor about your symptom changes or made a follow-up appointment to discuss these changes?
        1. *If yes*, what did you tell the doctor about your worsening symptoms, and what did s/he say in response?
        2. *If no,* why have you not told your doctor about your worsening symptoms or made an appointment to tell him/her?

________________________________________________________________________________________________________________________________________________________________________________________________________________________

________________________________________________________________________

________________________________________________________________________

1. Since the visit where we recorded you, has your physician made any changes to your [use patient term] medication? *(Note: Only include medication changes made after the visit, do not include changes made during the visit.)*

⁪ Yes ⁪ No

- 1. *If yes*, what change was made? (*Probe for dose change, an add or stop, & get the names of any meds changed.)*
     1. Why did s/he change your medication? (*Probe for symptom change, patient complaint, MD initiated, etc.*)
     2. Have you filled that prescription?
  2. *If no*, have you changed the way you take your medication? (*Probe if patient is still taking meds as instructed*)
     1. Are you satisfied with the medications in regards to your [use patient term]?

________________________________________________________________________________________________________________________________________________

________________________________________________________________________

________________________________________________________________________

1. Since the visit where we recorded you, have you stopped taking the medication for your [use patient term]?

⁪ Yes ⁪No

1. *If yes*, did you tell a healthcare professional this?

⁪ Yes ⁪No

- - 1. *If yes,* what did you tell the healthcare professional, and what did s/he say in response?
    2. *If no*, why did you stop taking it and not discuss this with a healthcare professional*?*

________________________________________________________________________________________________________________________________________________

________________________________________________________________________

________________________________________________________________________

1. Since the visit where we recorded you, have you seen any healthcare professionals for your [use patient term] other than the one we recorded you with?

⁪ Yes ⁪ No

- 1. *If yes,* what type of doctor did you see? (*Probe for PCP, GYN, URO)*
     1. What was the reason for seeing this doctor? (*Probe for referral from another MD, patient sought them out for a specific reason, etc.)*
  2. *If no,* under what circumstances, if any, would you anticipate needing to see a different doctor?

________________________________________________________________________________________________________________________________________________________________________________________________________________________

1. In the last 2 weeks, how many times, if at all, has your [use patient term] affected your daily routine or had an effect on how you feel (*probe for emotions)*? Please explain what effect it had on your daily routine.

# Of times: Impact

________________________________________________________________________________________________________________________________________________________________________________________________________________________

________________________________________________________________________

1. Have you and your doctor ever discussed the impact of your [use patient term] on your daily life?

⁪ Yes ⁪ No

1. *If yes*, when was the last time this was discussed?
   - 1. What was said?
2. *If no,* why do you think this has never been discussed?

________________________________________________________________________________________________________________________________________________________________________________________________________________________

________________________________________________________________________

*Interview conducted by: _____________________________________________*

*Date: ____________________________________________________________*

*Time: ____________________________________________________________*
