# Supplementary material for: Physician-patient communication about overactive bladder: Results of an observational sociolinguistic study
Source: PLoS One. 2017 Nov 15;12(11):e0186122. doi: 10.1371/journal.pone.0186122 (PMC5687746; doi:10.1371/journal.pone.0186122)
Supplement: S2 Text — (DOCX) [file pone.0186122.s002.docx]

*Thank you for your participation in our research. The purpose of our discussion today is to understand your journey with your urination issues. All your responses will be kept confidential and will not be shared with any of your physicians*.

| **Initial OAB Symptoms and Experiences [5-8 minutes]** |
| --- |

*I’m going to start off asking about when you first started experiencing problems with your bladder and urination, and then we will progress from there.*

1. When did you first start having problems with urination? (*Probe for age, or X many years ago, etc.)*
   1. Tell me about these problems. (*Probe for frequent urination or leakage*.)
   2. What do you call your urination issues? (*For example, “overactive bladder”, “frequent urination’, etc.)[****Please use patient terminology on how they describe their condition throughout interview****]*
   3. *If not mentioned by patient:* Have you heard the term overactive bladder?
2. When your symptoms first started, how did you **feel**? (*Probe for their* ***emotions,*** *i.e., frustrated, embarrassed, indifferent, etc.*)
   1. Did you discuss this with anyone besides your physician when you first noticed symptoms? *(Probe for friends, family, pharmacist, nurse, etc.)*
      1. *If yes*, what was discussed?
   2. Did you do any research when your symptoms started? (*Probe for internet, talk to friends/family, pharmacist, etc.)*.
3. Did you seek treatment at the first sign of problems or did you wait?
   1. *If immediate*: What made you decide to get care right away?
   2. *If patient waited*: How long did you wait?
      1. Why didn’t you see your doctor right away?
      2. In what circumstances would you see your doctor right away?

| **Initial OAB Diagnosis, OAB Medications [12-15 minutes]** |
| --- |

1. I’d like you to tell me about your experience the first time you talked about your [use patient term] with the doctor you visited with today. First, tell me how you felt during that first conversation.
   1. Did you make an appointment specifically to discuss your bladder problems or did it come up spontaneously during the visit?
   2. Who brought the topic up?
   3. Did you have any unanswered questions or concerns when leaving that visit?
   4. Since it was the first time you were discussing your [use patient term] with this doctor, was there anything you were holding back or did not want talk to the doctor about? (*Probe for if this was caused by their* ***emotions*** *i.e., embarrassment, fearful, hesitant, etc.*)
2. Did the doctor use any of the following terms when s/he discussed your bladder issues with you during the first conversation: *“overactive bladder,” “urge incontinence,” “urinary leakage,” or other*?
   1. How did you **feel** during this initial conversation?
3. What specific tests, if any, did the doctor use to diagnose you with your [use patient term]?
4. After you were initially diagnosed, did you begin medical treatment?
   1. *If yes*, what medication(s) did you start using?
   2. *If no*, why did you not begin treatment at this time?
      1. What did you do instead of beginning treatment? *(Probe to understand if medication was recommended, but they held off, and if so, why?)*
5. Are you currently using medication for your [use patient term]?
   1. *If yes*, are your current medications the same or different medication(s) than the ones you first started using for frequent urination?
      1. *If different*, what medications are you currently using now and what caused you to switch from [product name] to [product name]? (*Probe for all previous medications used for his/her [use patient term].*)
         1. How long were you taking each of your previous [use patient term] medications?
      2. *If the same*, do you have any concerns about your current mediations? (*Probe for specific concerns, if any.*)
         1. How long have you been taking this medication?
   2. *If no,* how is your [use patient term] being managed?
   3. *For all, ask:* Are you satisfied with how your [use patient term] is being managed?
6. Did you and your doctor discuss your current treatment for your [use patient term] today? (*If not mentioned, probe about generics for all follow-ups to this question.*)
   1. What do you recall from that discussion about your treatment?
   2. Did you and your doctor discuss any new treatment options today?
      1. *If yes*, tell me about the options that were discussed.
      2. How did you **feel** during this discussion with the doctor?

1. What did the doctor tell you about how your medications work? For example, did s/he tell you how well the medication would work or how long it would take before it started working?
   1. Did the doctor mention side effects, and if so, what did s/he say about them?
2. Did you and your physician ever discuss the cost of the medication?
   1. If so, what was discussed? (*Probe also for who brought this up.)*
   2. Did s/he mention if your medication would be a name brand or generic?
   3. Did the physician or any other staff members offer you samples? *(Probe for ever/today.)*
3. As a reminder, all your answers during this interview will be kept confidential—we will not share any of this with your doctor.

How often do you take your [use patient term] medication? (*Probe for everyday, sometimes I skip a dose, only as they feel like it/are able to, etc.*)

- 1. How has your doctor instructed you to take your [use patient term] medication (once a day, every other day, only when needed)?
  2. Have you ever stopped taking your [use patient term] medication without being instructed to do so by your doctor? (*Probe for never, sometimes, always*)
  3. *If yes*, why?
     1. What might be a reason for not taking your medication?
     2. How often does that happen? Daily, weekly, monthly or on occasion?
     3. Did you ever inform your doctor that you stopped taking this medication? If yes, what did the doctor say?
  4. Please take a moment and think about what, if anything, may help you to continue taking your medication on a daily basis.

| **Referral process [3 minutes]** |
| --- |

1. **If patient was seen by a PCP or OBGYN today:**

Have you ever been referred to a urologist for your [use patient term]?

[*If patient has never been referred to a urologist, skip to question 14.*]

- 1. What was that experience like?
  2. How would you characterize the difference in the treatment between your PCP/OBGYN and the urologist?
  3. How would you characterize the difference in the discussion between your PCP/OBGYN and the urologist?

**If patient was seen by a urologist today:**

1. What was the experience like being referred from your PCP/OBGYN to the urologist?
2. Do you recall for what specific reason you were referred? (*Probe for a specific event, or symptom, or test, etc.*)
3. How would you characterize the difference in the treatment and in the discussion between the PCP/OBGYN and the urologist?
4. How comfortable were you with talking to someone other than your regular doctor?

| **current OAB discussions [3-4 minutes]** |
| --- |

1. I’d like to talk a little about the discussions you now have with your doctor about your [use patient term]. Do you discuss your [use patient term] at each visit?
   1. *If not*, how often do you discuss it? *(Probe for duration or situation, such as when I need a medication refill.)*
   2. Do you ever make follow-up appointments specifically to talk about your [use patient term] or do you wait until your next scheduled appointment?
      1. *If patient waits*: Why do you wait to make an appointment? What would make you schedule an appointment vs. wait?
      2. *If patient makes appointments*: What circumstances lead you to make specific appointments?
2. What were the 3 most important things you told the doctor about your [use patient term] today?
   1. What **thoughts/emotions** stand out when you were telling the doctor these things?
   2. Did the doctor appear to understand what you were telling him/her? Please explain.
   3. Was there anything you wanted to talk to the doctor about but did not? (*Probe for what and why it was not discussed.*)
3. When you talk about your symptoms what does the doctor say?
   1. What term or phrase does s/he use?
   2. How do you **feel** about how the doctor addresses your condition?

| **Quality of Life, Goals, and Perception of Future [3-4 minutes]** |
| --- |

1. How does your [use patient term] affect your life?
   1. Do you discuss that with the doctor?
2. What are your goals for your [use patient term] treatment?
3. When you think about your future with your [use patient term], what do you see? (*Probe for whether they think it will get better or worse.*)
   1. Will you continue treating with this medication or do you think you will switch? Please explain.
   2. When are you planning to see the doctor next for [use patient term]?
4. Please take a moment to think about what you want from your [use patient term] treatment, and then complete this sentence for me: “If only I had a treatment that would make me **feel** [blank]…*…*then I could [blank]…” *(Probe for why they used those words or phrases.)*
