# Supplementary material for: Physician-patient communication about overactive bladder: Results of an observational sociolinguistic study
Source: PLoS One. 2017 Nov 15;12(11):e0186122. doi: 10.1371/journal.pone.0186122 (PMC5687746; doi:10.1371/journal.pone.0186122)
Supplement: S3 Text — (DOCX) [file pone.0186122.s003.docx]

| First Name, Last Initial: _____________________________________________ __________  Doctor Name: _______________________________ Date: _________________________ |
| --- |

*Thank you for taking the time to participate in this research. Please answer the following questions with a 🗹 in the appropriate box and/or a written answer where indicated (4 pages):*

| 1. | | What is your age? |  | ________________________________ ____ | |
| --- | --- | --- | --- | --- | --- |
| 2. | | What is your gender? | ⁭  ⁭ | Female  Male | |
| 3. | | When did you first begin experiencing problems with your bladder? |  | Month_______________ Year________________ | |
| 4. | | Have you ever been diagnosed with overactive bladder or frequent urination by your doctor? | ⁭  ⁭ | Yes  No  ***If yes, when***:  Month_______________ Year________________ | |
| 5. | | How long have you been a patient of this doctor? | ⁭  ⁭  ⁭  ⁭  ⁭ | This is my first visit  Less than 1 year (specify # of months):___ _____  Between 1 and 3 years  Between 3 and 5 years  More than 5 years | |
| 6. | | In the past 12 months, how often have you seen this doctor? | ⁭  ⁭  ⁭  ⁭  ⁭  ⁭  ⁭ | This is my first visit  More than once per month  Once per month  Every 2-3 months  Every 4 months  Every 6 months  Other (specify) ______________ ______ | |
| 7. | What was the purpose of your visit ***today***? | | ⁭  ⁭  ⁭  ⁭ | Routine Visit  Follow-up visit for your condition(s)  Complications from treatment/medications  Other______ _____________________ | |
| 8. | Are you currently using medication for your bladder? | | ⁭  ⁭ | Yes  No (***If no, please skip to question 10***) | |
| 9. | How satisfied are you with your ***current*** treatment for your bladder? | | ⁭  ⁭  ⁭ | Extremely satisfied  Satisfied  Unsatisfied | |
| 10. | Do you plan to follow the recommendations your doctor made ***today*** about treatment for your bladder issues? | | ⁭  ⁭ | Yes  No | |
| 11. | To what degree were the questions you wanted to discuss addressed ***today***? | | ⁭  ⁭  ⁭ | Addressed completely  Addressed somewhat  Not addressed at all | |
| 12. | Did you find your communication with the doctor successful ***today***? | | ⁭  ⁭ | Yes  No | |
| 13. | How satisfied are you with ***this*** doctor in general? | | ⁭  ⁭  ⁭ | Extremely satisfied  Moderately satisfied  Unsatisfied | |
| 14. | Which of these best describes the role you and/or your friends/family play vs. the doctor’s role in making decisions about your health? | | ⁭  ⁭  ⁭  ⁭ | I/we are the most actively involved  My doctor and I/we participate equally  Although I/we are involved, my doctor is more responsible  I/we are not involved; I have complete faith in my doctor’s decisions | |
| 15. | Is there a nurse practioner, physician assisnant, nurse, or other allied health care practitioner who is involved in your treatment decisions? | | ⁭  ⁭  ⁭  ⁭  ⁭ | Yes, Nurse practitioner  Yes, Physician assistant  Yes, Nurse  Yes, Other (***specify)*** _________________  ***Please briefly describe each person’s role:***  ___________________________________  ___________________________________  ___________________________________  No | |
| 16. | When your doctor recommends a treatment to you, what do you typically do? | | ⁭⁭  ⁭  ⁭ | I always follow recommendations faithfully  I sometimes follow my doctor’s recommendations  I sometimes follow my doctor’s recommendations ***but only after*** I do my own research  I hardly ever follow recommendations | |
| 17. | Which best describes your level of concern for your bladder issues? | | ⁭  ⁭  ⁭ | I am very concerned  I am somewhat concerned  I never worry | |
| 18. | | Do you have health insurance?  (***Check all that apply***) | ⁭  ⁭  ⁭  ⁭  ⁭  ⁭  ⁭  ⁭ | Yes, private insurance (e.g., Aetna, Cigna)  Yes, Medicare  Yes, Medicaid  Yes, Veterans’ Administration/other military  Yes, Worker’s Comp  Yes, COBRA  Yes, other (specify) ______________ ___  No |  |
| 19. | | Do you have prescription drug coverage?  (***Check all that apply***) | ⁭  ⁭  ⁭  ⁭  ⁭  ⁭ | Yes, private insurance (e.g., Aetna, Medco)  Yes, Medicare Part D  Yes, Medicaid  Yes, Worker’s Comp  Yes, other (specify) ____________________  No |  |
| 20. | For reporting purposes, can you tell me which best describes your ethnicity? | | ⁭  ⁭  ⁭  ⁭  ⁭  ⁭  ⁭  ⁭ | Caucasian  Asian (including Indian subcontinent)  African-American  Hispanic  Middle Eastern  Pacific Islander/Native American  Other, please specify: ____ __________  I do not wish to provide this information |  |
| 21. | What is the highest level of education you have completed? | | ⁭  ⁭  ⁭  ⁭  ⁭  ⁭  ⁭ | High School  Some College  Associate Degree  Bachelor’s Degree  Advanced Degree  Other_________________________ ___  I do not wish to provide this information |  |

*lease indicate any other conditions that you have, and what medication, if any, you are taking as treatment for that condition.*

| **Condition:** | **Do you have this condition?** | | **If yes, please indicate any medication(s) taken for it** |
| --- | --- | --- | --- |
| ***EXAMPLE****:* |  |  |  |
| *Allergies/hay fever* | ‏‏‏🗹 Yes | □ No | *Allegra-D* |
| Acid reflux | ‏□ Yes | □ No | __________________________________ |
| Allergies/hay fever | ‏□ Yes | □ No | __________________________________ |
| Anxiety | □ Yes | □ No | __________________________________ |
| Arthritis, Osteoarthritis, Rheumatoid  arthritis and unspecified | □ Yes | □ No | __________________________________ |
| Asthma | □ Yes | □ No | __________________________________ |
| Cancer | □ Yes | □ No | __________________________________ |
| Chest pain/pressure (angina) | □ Yes | □ No | __________________________________ |
| Chronic Fatigue Syndrome | □ Yes | □ No | __________________________________ |
| COPD/emphysema | □ Yes | □ No | __________________________________ |
| Depression | □ Yes | □ No | __________________________________ |
| Diabetes | □ Yes | □ No | __________________________________ |
| Epilepsy (seizures) | □ Yes | □ No | __________________________________ |
| Fibromyalgia | □ Yes | □ No | __________________________________ |
| Glaucoma | □ Yes | □ No | __________________________________ |
| Heart attack | □ Yes | □ No | __________________________________ |
| Heart disease | □ Yes | □ No | __________________________________ |
| High cholesterol | □ Yes | □ No | __________________________________ |
| High blood pressure/hypertension | □ Yes | □ No | __________________________________ |
| HIV/AIDS | □ Yes | □ No | __________________________________ |
| Irregular heartbeat | □ Yes | □ No | __________________________________ |
| Insomnia/sleep disorder | □ Yes | □ No | __________________________________ |
| Kidney disease | □ Yes | □ No | __________________________________ |
| Lupus | □ Yes | □ No | __________________________________ |
| Migraine headaches | □ Yes | □ No | __________________________________ |
| Multiple Sclerosis | □ Yes | □ No | __________________________________ |
| Sleep apnea | □ Yes | □ No | __________________________________ |
| Stroke | □ Yes | □ No | __________________________________ |
| Thyroid disease | □ Yes | □ No | __________________________________ |
| Other (specify) _____________ | □ Yes | □ No | __________________________________ |
| Other (specify) _____________ | □ Yes | □ No | __________________________________ |
| Other (specify) _____________ | □ Yes | □ No | __________________________________ |

*THANK YOU FOR YOUR PARTICIPATION!*
