# Supplementary material for: Physician-patient communication about overactive bladder: Results of an observational sociolinguistic study
Source: PLoS One. 2017 Nov 15;12(11):e0186122. doi: 10.1371/journal.pone.0186122 (PMC5687746; doi:10.1371/journal.pone.0186122)
Supplement: S4 Text — (DOCX) [file pone.0186122.s004.docx]

*Thank you for your participation in our research. The purpose of our discussion today is to understand your perspective on treating patients with overactive bladder. All your responses will be confidential and will not be shared with your patients.*

| **General questions regarding OAB treatment & referral [12-15 minutes]** |
| --- |

*First I would like to talk generally about your approach to overactive bladder and its treatment. Then I will get into a set of specific questions that I will ask for each patient.*

1. How do you typically diagnose overactive bladder (OAB)? Are there symptoms you specifically look for and/or diagnostic tests you perform?
   1. What language (words or phrases) do you hear from patients that helps you distinguish between urinary leakage, urge incontinence, overactive bladder and stress incontinence?
2. How do you treat overactive bladder? (*Probe for the variables the physician uses to make a choice*).
   1. Do you typically start a patient on a prescription medication upon diagnosis?
      1. If not, why not?
   2. Do you ever recommend non-pharmacologic treatment?
3. How often do you believe patients stop treatment for OAB prior to being instructed to do so by his/her physician? (*Probe for never, sometimes, always*)
   1. What are the reasons patients typically stop treatment for OAB?
4. Do you ever stop patients on prescription medication for OAB? *If yes*, for what reasons?
5. *Now I would like to discuss the referral process for OAB*

**If interviewing a PCP or OBGYN:**

- 1. At what point, clinically speaking, do you decide to refer a patient to a urologist?
  2. What percentage of the time do you refer patients to a urologist for OAB treatment as opposed to treating them yourself?
  3. What, if anything, does referral to a urologist offer an OAB patient beyond the treatment you provide?

**If interviewing a urologist:**

- 1. What percent of your OAB patients come directly to you due to symptoms as opposed to being referred from a PCP or OBGYN?
     1. Do most of your referrals for OAB come from PCPs or OBGYNs?

1. How would you characterize the difference between how a PCP approaches OAB treatment versus a urologist?
2. How would you characterize the difference between how an OBGYN approaches OAB treatment versus a urologist?
3. Do you continue to manage a referred patient’s OAB or do you send the patient back to the PCP or OGBYN to manage them on an ongoing basis?

| **Initial OAB Symptoms and Experiences [5-8 minutes]** |
| --- |

*Now I would like to ask questions about each individual patient. I’m going to start off asking about when [patient name] first started experiencing problems with overactive bladder, and we will progress from there.*

1. When did [patient name] first start having problems with overactive bladder? (*Probe for age, or X many years ago, etc.)*
   1. Does this patient have urge incontinence symptoms only, stress incontinence symptoms only, or a mix of both?
      1. *If physician says stress incontinence only*, what makes you think this patient no longer has urge incontinence? (*Probe for symptom changes, etc.)*
2. Did this patient seek treatment at the first sign of problems or did they wait?
   1. *If immediate*: Do you know what made them decide to get care right away?
   2. *If patient waited*: Do you know why they didn’t seek care right away?

| **Initial OAB Diagnosis, OAB Medication [12-15 minutes]** |
| --- |

1. When was this patient first diagnosed with overactive bladder (*probe for age, X many years ago, etc.*)?
   1. What type of doctor told him/her the diagnosis? (*Probe for PCP, OBGYN, URO, etc.)*
2. I’d like you to tell me about the first time you talked about overactive bladder with [patient name]. Did he/she make an appointment specifically to discuss his/her bladder problems or did it come up spontaneously during a visit?
   1. Who brought the topic up?
   2. Did you use any of the following terms when you discussed his/her overactive bladder: *“overactive bladder,” “urge incontinence,” “urinary leakage,” or other*?
      1. How did you think the patient **felt** during this conversation?
3. What specific tests, if any, were used to diagnose this particular patient with overactive bladder?
4. After [patient name] was initially diagnosed, did you start him/her on pharmacologic treatment?
   1. *If yes*, what medication(s) did you start him/her on?
      1. Why did you choose these particular treatments?
      2. What did you tell the patient to expect from these treatments, in terms of benefits, side effects, length of therapy?
      3. Was the patient given samples at this time?
   2. *If no*, why did the patient not begin treatment at this time?
      1. What did you decide to do instead of beginning treatment? (*Probe to understand if medication was recommended, but they held off, and if so, why?)*
5. Is the patient currently using medication for his/her overactive bladder?
   1. *If yes*, is the patient using the same or different medication(s) now than the ones he/she first started using for overactive bladder?
      1. *If different*, what are they using now and why did you switch from [Product X] to [Product Y]? (*Probe for all previous OAB meds.*)
      2. *If the same,* does the patient have any concerns about their medications?
   2. *If no*, how is their overactive bladder being managed?
6. Did you and [patient name] discuss his/her current treatment for overactive bladder today? (*If not mentioned, probe about generics for all follow-ups to this question.*)
   1. What did you tell the patient about their treatment?
7. Did you and [patient name] discuss any new treatment options for his/her overactive bladder today?
   1. *If yes*, what options were discussed?
   2. What did you tell the patient about those options?
      1. How did you think the patient **felt** during that conversation?
8. What did you tell the patient about how his/her overactive bladder medications work? For example, did you explain how well the medication would work or how long it would take before they started working?
   1. Did you mention side effects, and if so, what have you said say about them?
9. Did you ever discuss with this patient the cost of overactive bladder medication?
   1. If so, what was discussed? (*Probe also for who brought this up.)*
   2. Did you mention if the medication would be a name brand or generic?
   3. Did you or any other staff members offer this patient samples? *(Probe for ever/today.)*
10. Has this patient ever mentioned that he/she has discontinued taking his/her overactive bladder medication without being instructed to do so by you?
    1. *If yes*, do you know why he/she stopped?
       1. How long after he/she stopped did you find out about it?
       2. What did you say when you found out?
    2. Please take a moment and think about what, if anything may help *this patient* to continue taking his/her medication on a daily basis.

| **Referral process [3 minutes]** |
| --- |

1. **If interviewing a PCP or OBGYN today:**

Have you ever referred this patient to a urologist for overactive bladder?

- 1. *If yes*, for what reason were they referred?
  2. *If no*, under what circumstances would you refer them?

**If interviewing a urologist today:**

1. Do you recall for what reason this patient was referred to you? (*Probe for a specific event, or symptom, or test, etc.*)

| **current OAB discussions [3-4 minutes]** |
| --- |

1. I’d like to talk a little about the discussions you now have with [patient name] about overactive bladder. Do you discuss his/her overactive bladder at each visit?
   1. *If not*, how often do you discuss it? *(Probe for duration or situation, such as when patient needs a medication refill.)*
   2. If this patient does not mention his/her overactive bladder during a regular appointment, do you ask him/her about it?
      1. Do you ask [patient name] if he/she is still taking the medications?
2. Does [patient name] ever make follow-up appointments specifically to talk about overactive bladder or does he/she wait until his/her next scheduled appointment?
   1. *If patient waits*: Why does he/she wait to make an appointment?
   2. *If patient makes appointments:* Why does he/she make appointments?
3. What were the 3 most important things the patient told you about his/her OAB today?
   1. What **thoughts/emotions** did the patient express when s/he was telling you that information?
   2. What were your **thoughts/emotions** when s/he was telling you that information?
4. When you discuss [patient name’s] symptoms, what does he/she say?
   1. Do they have a preferred term or phrase?
   2. What is their **emotional** state when they discuss it? (*Probe for* ***emotions*** *e.g. embarrassed, hesitant, etc.*)
   3. Does this patient tend to discuss specific symptoms or overall impact on quality of life?
5. What do you need to hear or see from a patient to help with treatment or diagnosis?
   1. Are there words/phrases, symptoms, concerns, etc. that stand out to help you in diagnosis?
   2. Is there a particular **emotion** or **feeling** that they express or that you observe that helps?

| **Quality of Life, Goals, and Perception of Future [3-4 minutes]** |
| --- |

1. How does overactive bladder affect [patient name’s] life?
   1. Is that something you and the patient have discussed before?
2. What are your goals for this patient’s overactive bladder treatment?
   1. What do you believe are the patient’s goals?
3. When you think about [patient name’s] future with overactive bladder, what do you see? (*Probe for whether they think it will get better or worse.*)
   1. Will you continue treating with this medication or do you think you will switch? Please explain.
   2. When do you think you will see this patient next for overactive bladder?
4. Please complete this sentence for me: “If only I had a treatment that would make my patient **feel** [blank]…” *(Probe for why they us****e****d those words or phrases.)*
