# Supplementary material for: Physician-patient communication about overactive bladder: Results of an observational sociolinguistic study
Source: PLoS One. 2017 Nov 15;12(11):e0186122. doi: 10.1371/journal.pone.0186122 (PMC5687746; doi:10.1371/journal.pone.0186122)
Supplement: S5 Text — (DOCX) [file pone.0186122.s005.docx]

| Patient’s First Name, Last Initial______________________________________________  Doctor Name: _______________________________ Date: _______________________ |
| --- |

*Thank you for taking the time to participate in this research. Please answer the following questions with a 🗹 in the appropriate box and/or a written answer where indicated (2 pages).*

| 1. | When did this patient first begin experiencing problems with overactive bladder or frequent urination? |  | Month ______________ _  Year ________________ | |
| --- | --- | --- | --- | --- |
| 2. | Has this patient been diagnosed with overactive bladder? | ⁭  ⁭ | Yes  No  ***If yes, when***:  Month ____________ _ __  Year _____________ ___ | |
| 3. | How long has this individual been a patient of yours? | ⁭  ⁭  ⁭  ⁭  ⁭ | This is his/her first visit  Less than 1 year: # of months: ___________  Between 1 and 3 years  Between 3 and 5 years  More than 5 years | |
| 4. | How often does this patient come in for regularly scheduled visits? | ⁭  ⁭  ⁭  ⁭  ⁭  ⁭  ⁭ | This is his/her first visit  More than once per month  Once per month  Every 2-3 months  Every 4 months  Every 6 months  Other (specify) _______________________ | |
| 5. | What was the purpose of this patient’s visit ***today***? | ⁭  ⁭  ⁭  ⁭ | Routine visit  Follow-up visit for symptom(s)  Complications with treatment/medications  Other_______________________________ | |
| 6. | Is this patient ***currently*** using medication for overactive bladder? | ⁭  ⁭ | Yes  No (***If no, please skip to question 9***) | |
| 7. | How satisfied are you with this patient’s ***current*** treatment for overactive bladder? | ⁭  ⁭  ⁭ | Extremely satisfied  Satisfied  Unsatisfied | |
| 8. | Do you think the patient will follow the recommendations you made ***today*** about treatment? | ⁭  ⁭ | Yes  No | |
| 9. | To what degree were the topics you wanted to discuss addressed ***today***? | ⁭  ⁭  ⁭ | Addressed completely  Addressed somewhat  Not addressed at all | |
| 10. | Did you find your communication with the patient successful ***today?*** | ⁭  ⁭ | Yes  No | |
| 11. | Do you think this patient would recommend you to his/her family/friends? | ⁭  ⁭ | Yes  No | |
| 12. | Which of these best describes ***your*** role versus the patient’s role when it comes to making decisions about his/her treatment? | ⁭  ⁭  ⁭  ⁭ | The patient is the person most actively involved in treatment decisions  The patient and I participate equally in treatment decisions  Although S/he is involved, I am more responsible for treatment decisions  The patient is not involved in these decisions; S/he has complete faith in me | |
| 13 | Is there an NP, PA, RN, or other allied health care practitioner involved in treatment decisions with this patient? | ⁭  ⁭  ⁭  ⁭  ⁭ | Yes, NP  Yes, PA  Yes, RN  Yes, Other (***specify)*** _________________  ***Please briefly describe each person’s role:***  ___________________________________  ___________________________________  ___________________________________  No | |
| 14. | Which of these best describes this patient’s habits with regard to following treatment recommendations? | ⁭  ⁭  ⁭ | S/he always follow recommendations faithfully  S/he sometimes follows my recommendations  S/he sometimes follows my recommendations ***but only after*** s/he does their own research  S/he hardly ever follows recommendations | |
| 15. | Which best describes this patient’s level of concern for his/her overactive bladder? | ⁭  ⁭  ⁭ | S/he is very concerned  S/he is somewhat concerned  S/he never worries | |
| 16. | What specific products for overactive bladder were prescribed or given as samples today? (list all names)?  ***Please specify whether each product is a new Rx, refill, or sample.*** |  | ___________________________________  ___________________________________  ___________________________________  ___________________________________ | |
| 17. | Did you discuss a change in this patient’s overactive bladder medication today? | ⁭  ⁭ | Yes   1. Adding a new medication; (**please specify which medication**) __________________ 2. Stopping one S/he currently uses; (**please specify which medication**) ___________________   No |  |
| 18. | Does this patient have health insurance?  (***Check all that apply***) | ⁭  ⁭  ⁭  ⁭  ⁭  ⁭  ⁭  ⁭ | Yes, private insurance (e.g., Aetna, Cigna)  Yes, Medicare  Yes, Medicaid  Yes, Veterans’ Administration/other military  Yes, Worker’s Comp  Yes, COBRA  Yes, other (specify) _____________ ____  No |  |
| 19. | Does this patient have prescription drug coverage?  (***Check all that apply***) | ⁭  ⁭  ⁭  ⁭  ⁭  ⁭ | Yes, private insurance (e.g., Aetna, Medco)  Yes, Medicare Part D  Yes, Medicaid  Yes, Worker’s Comp  Yes, other (specify) ____________________  No |  |
| 20. | Does the patient’s financial or insurance status/coverage factor into medication decisions? | ⁭  ⁭ | Yes, ***please explain***: __________________________________________________________________________  No |  |
| 21. | For reporting purposes, can you tell me which best describes your ethnicity? | ⁭  ⁭  ⁭  ⁭  ⁭  ⁭  ⁭  ⁭ | Caucasian  Asian (including Indian subcontinent)  African-American  Hispanic  Middle Eastern  Pacific Islander/Native American  Other, please specify: ______________  I do not wish to provide this information |  |

*THANK YOU FOR YOUR PARTICIPATION!*
